# Supplementary material for: Interest of phenomic prediction as an alternative to genomic prediction in grapevine
Source: Plant Methods. 2022 Sep 5;18:108. doi: 10.1186/s13007-022-00940-9 (PMC9442960; doi:10.1186/s13007-022-00940-9)
Supplement: Supplementary file 1 — Additional file 1: Figure S1. Heritability distribution across wavelengths for each pre-process. Figure S2. Co-inertia analysis between SNP and multi-year wood and leaves NIRS matrices. Figure S3. Co-inertia analysis between SNP, single and multi-year wood and leaves NIRS matrices. Figure S4. Distribution of phenomic prediction predictive ability per pre-process and per trait. Figure S5. Predictive ability of phenomic prediction with a single vs both tissues. Figure S6. Comparison of methods for genomic prediction. Figure S7. Distribution of phenomic prediction predictive ability over 15 traits in each half-diallel cross. Figure S8. Predictive ability of combined vs genomic prediction models. [file 13007_2022_940_MOESM1_ESM.pdf]

Supplementary information

Interest of phenomic prediction as an alternative to genomic prediction in grapevine

Charlotte Brault[1,2,3], Juliette Lazerges[1,2], Agnès Doligez[1,2], Miguel Thomas[1,2], Martin Ecarnot[1], Pierre Roumet[1], Yves Bertrand[1,2], Gilles Berger[1,2], Thierry Pons[1,2], Pierre François[1,2], Loïc Le Cunff[2,3], Patrice This[1,2], Vincent Segura[1,2,*]

[1] AGAP Institut, Univ Montpellier, CIRAD, INRAE, Institut Agro, Montpellier, France

[2] UMT Geno-Vigne®, IFV-INRAE-Institut Agro, Montpellier, France

[3] Institut Français de la Vigne et du Vin, Montpellier, France

[*] correspondance : vincent.segura@inrae.fr

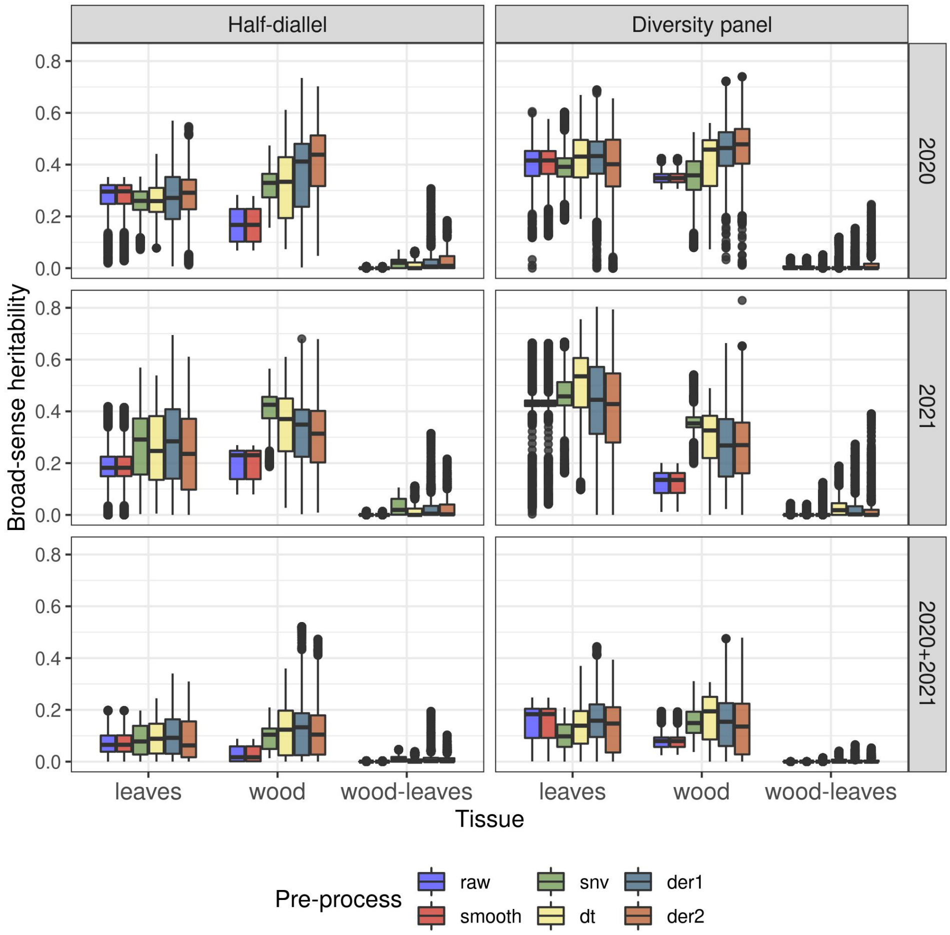

Figure S1 Heritability distribution across wavelengths for each pre-process

Comparison of broad-sense heritability between the six NIRS pre-processes, displayed for both populations, three year and three tissue BLUP models.

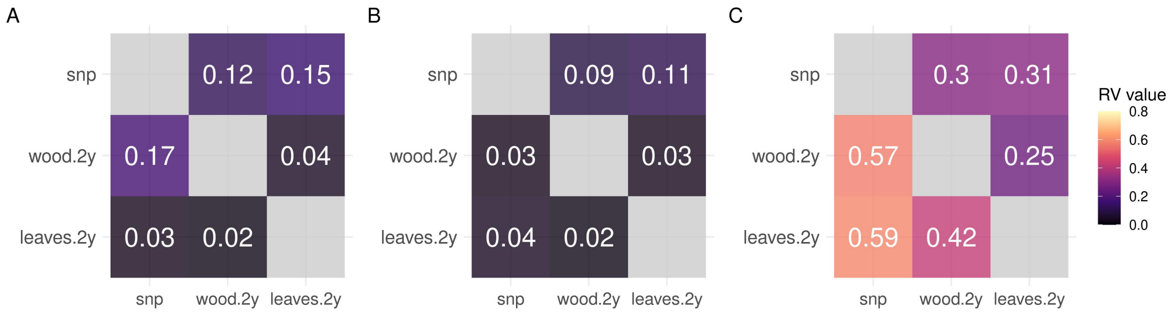

Figure S2 Co-inertia analysis between SNP and multi-year wood and leaves NIRS matrices

Upper triangle: in the diversity panel, lower triangle: in the half-diallel

A: mixed model with only genotype effect, resulting NIRS matrix of genotype BLUPs

B mixed model with genotype and *subpopulation* or *cross* effects, resulting NIRS matrix of genotype BLUPs only

C: mixed model with genotype and *subpopulation* or *cross* effects, resulting NIRS matrix of genotype + *subpopulation* or *cross* BLUPs

All mixed models were fitted after der1 pre-process.

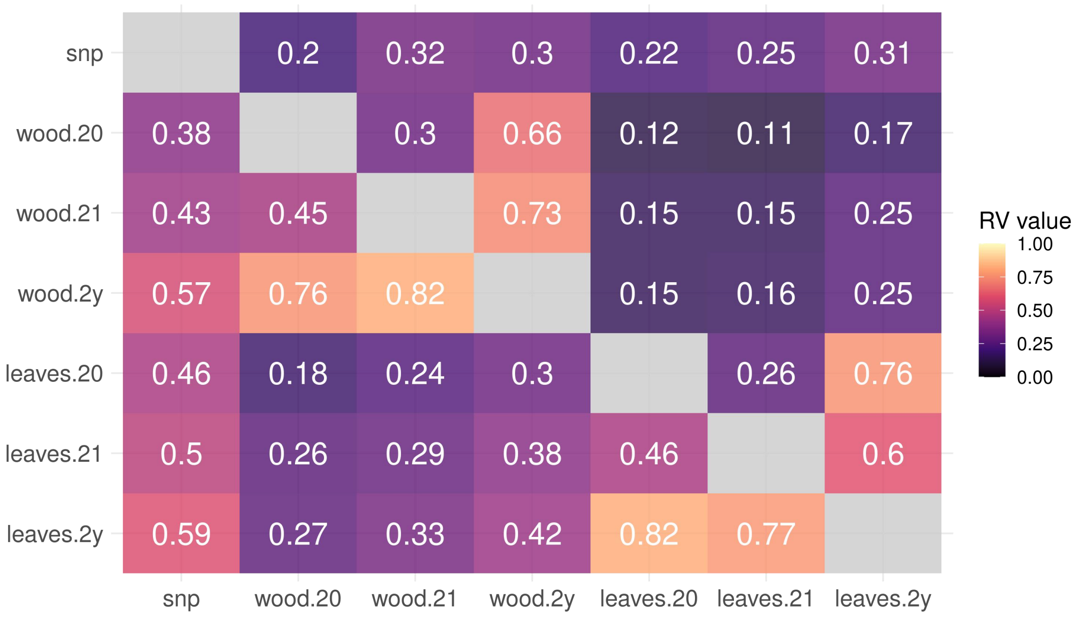

Figure S3 Co-inertia analysis between SNP, single and multi-year wood and leaves NIRS matrices

Upper triangle: in the diversity panel, lower triangle: in the half-diallel
Suffixes “20”, “21”, “2y” correspond to 2020, 2021 and multi-year NIRS, respectively. All mixed models were fitted after der1 pre-process.

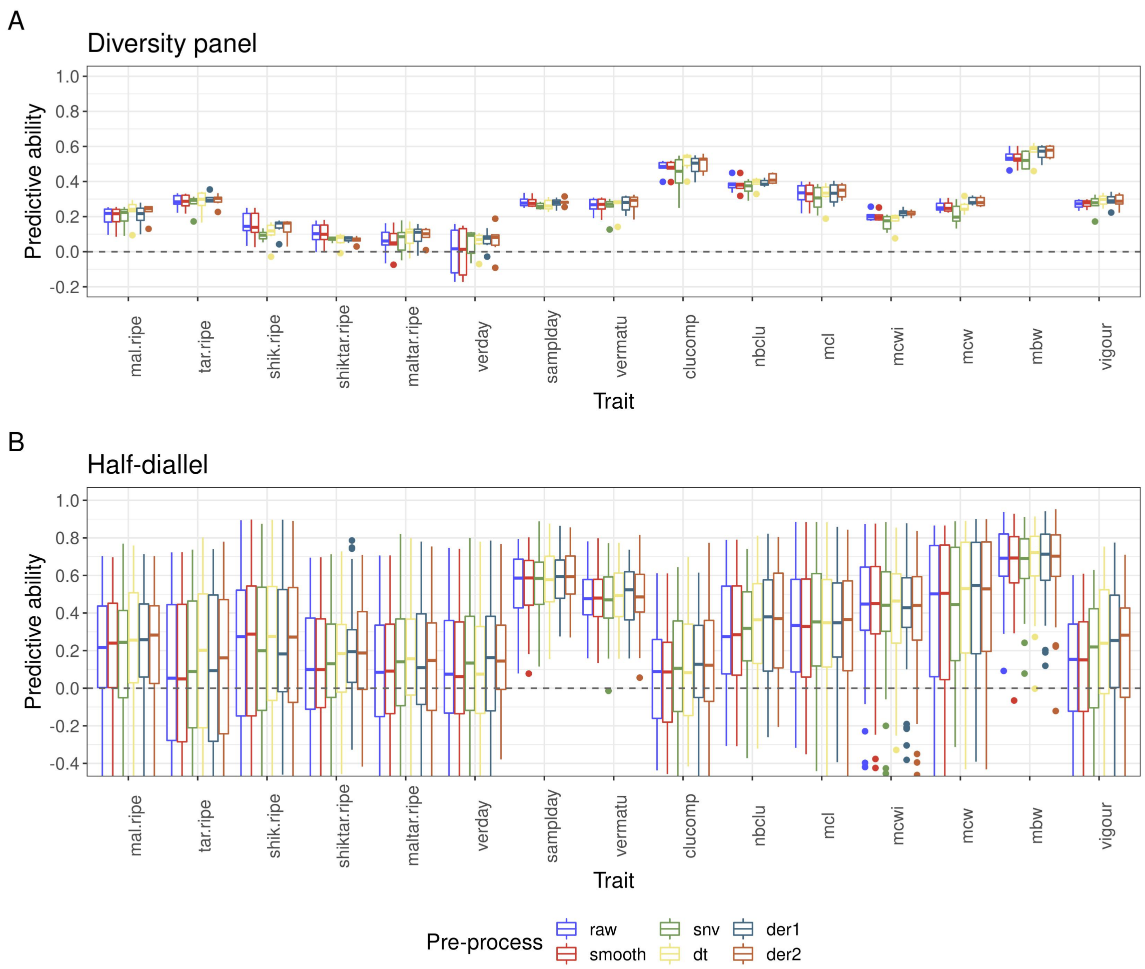

Figure S4 Distribution of phenomic prediction predictive ability per pre-process and per trait

A: in the diversity panel with rrBLUP method (implemented with glmnet); B: in the half-diallel with HBLUP method (implemented with lme4GS).

Distribution of predictive ability is displayed across two tissue x three year BLUP models, times ten crosses in the half-diallel.

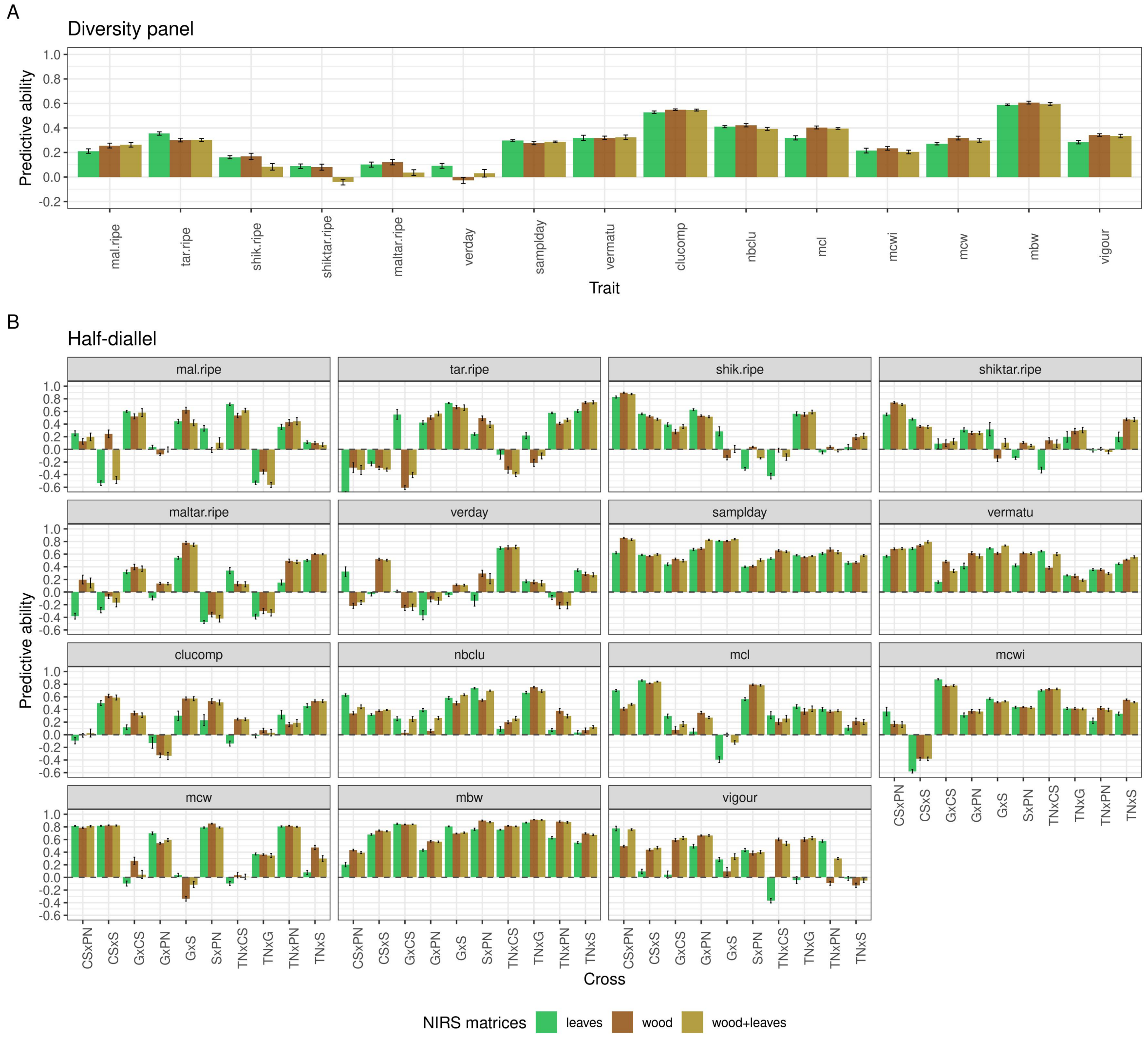

Figure S5 Predictive ability of phenomic prediction with a single vs both tissues

For “2 years” NIRS BLUPs derived after der1 pre-process. A: in the diversity panel, B: in the half-diallel. Predictive ability values are displayed per trait for both populations, and also per cross in the half-diallel. Prediction models were fitted with glmnet in the diversity panel (except for wood+leaves configuration) and with lme4GS in the half-diallel. Error bars correspond to 95% confidence intervals around the mean, calculated for the ten CV repetitions.

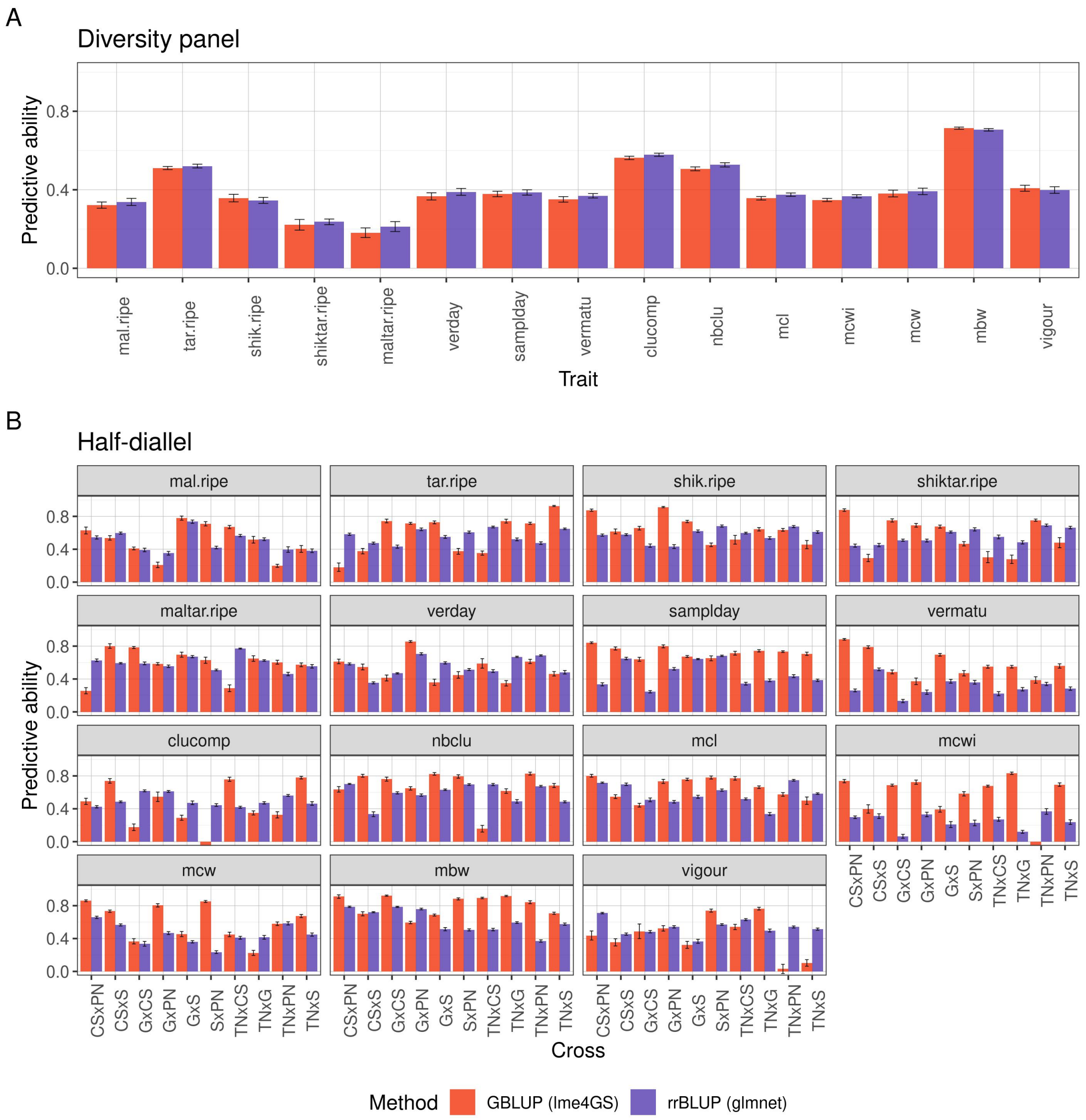

Figure S6 Comparison of methods for genomic prediction

A: in the diversity panel; B: in the half-diallel. Predictive ability values are displayed per trait for both populations, and also per cross in the half-diallel. Error bars correspond to 95% confidence intervals around the mean, calculated for the ten CV repetitions.

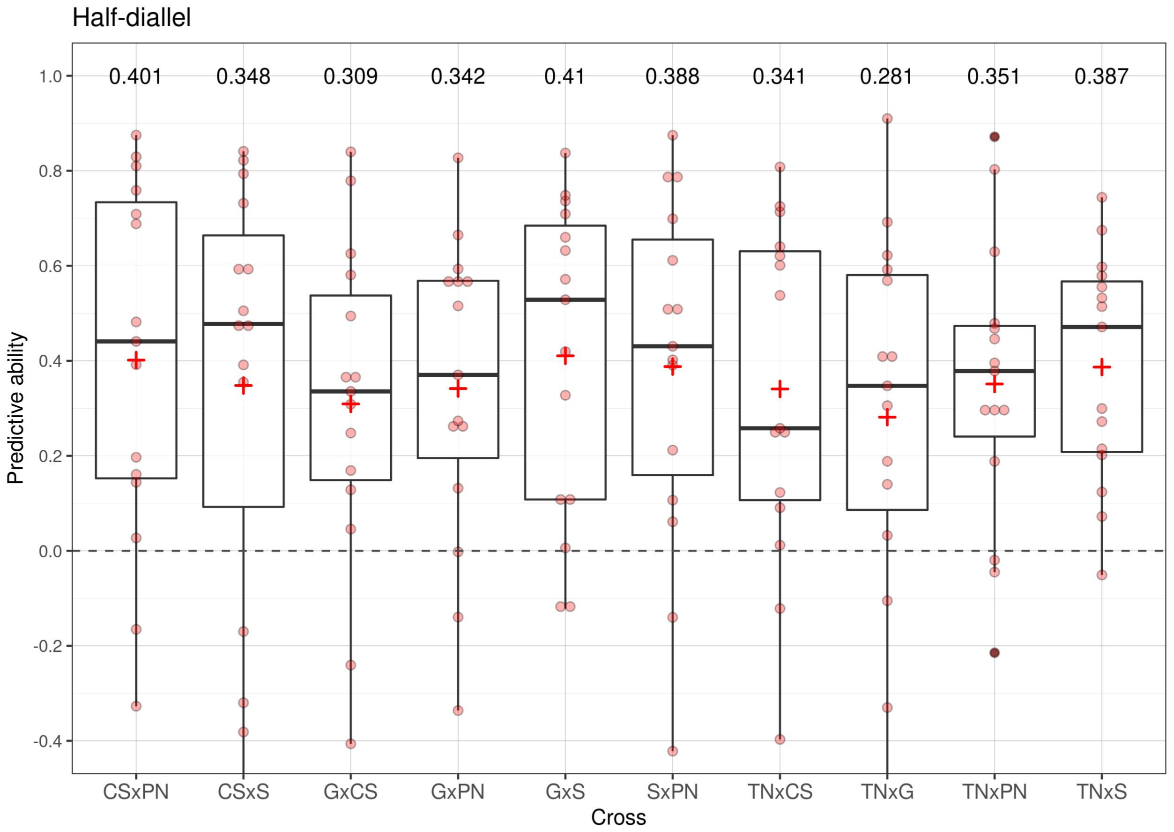

Figure S7 Distribution of phenomic prediction predictive ability over 15 traits in each half-diallel cross

For “2 years” NIRS BLUPs derived after der1 pre-process. Average PA per cross is displayed above each cross and with a red cross. Prediction models were fitted with lme4GS and included both wood and leaves NIRS relationship matrices.

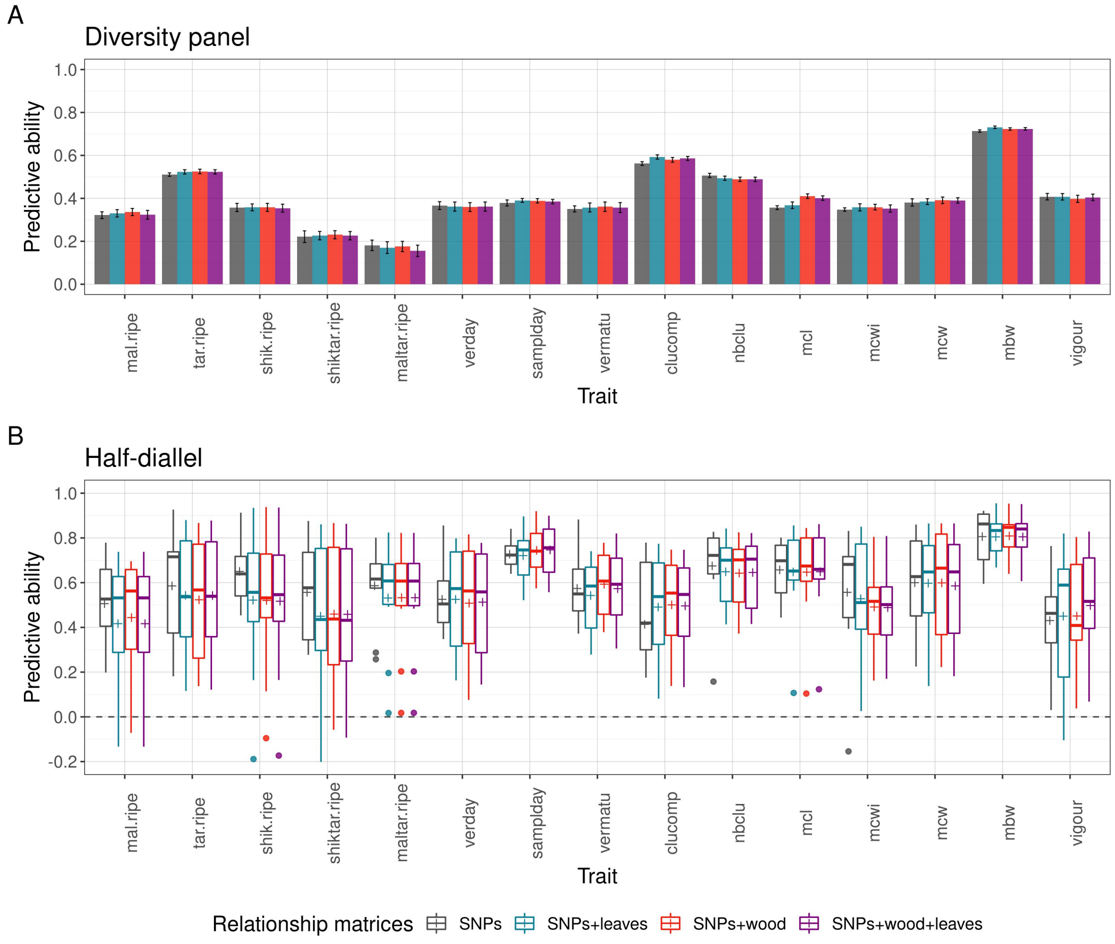

Figure S8 Predictive ability of combined vs genomic prediction models

A: in the diversity panel (error bars correspond to 95% confidence intervals around the mean, calculated for the ten CV repetitions); B: in the half-diallel (distribution over 10 crosses and 10 CV repetitions). Prediction models were fitted with lme4GS and included as relationship matrices: SNPs, SNPs and leaves NIRS, SNPs and wood NIRS or SNPs, wood and leaves NIRS. Wood and leaves NIRS BLUPs were derived from a mixed model including both years, after der1 pre-process
